# Novel Molecular Biomarkers at the Blood-Brain Barrier in ALS

**DOI:** 10.1155/2014/907545

**Published:** 2014-05-11

**Authors:** Danijela Bataveljic, Milena Milosevic, Lidija Radenovic, Pavle Andjus

**Affiliations:** Center for Laser Microscopy, Institute for Physiology and Biochemistry, Faculty of Biology, University of Belgrade, Studentski Trg 16, POB 52, 11000 Belgrade, Serbia

## Abstract

Recently neuroinflammation has gained a particular focus as a key mechanism of ALS. Several studies* in vivo* as well as* in vitro *have nominated immunoglobulin G (IgG) isolated from ALS patients as an active contributor to disease onset and progression. We have shown that ALS IgG affects astroglial Ca^2+^ excitability and induces downstream activation of phosphatidylinositol 3-kinase. These studies were hampered by a lack of knowledge of the pathway of entry of immune factors in the CNS. Our MRI data revealed the blood-brain barrier BBB leakage and T cell infiltration into brain parenchyma in ALS G93A rats. Since astrocyte ensheathes blood vessel wall contributing to BBB stability and plays an important role in ALS pathogenesis, we have studied astrocytic membrane proteins water channel aquaporin-4 and the inwardly rectifying potassium channel. In this review, we will summarize data related to BBB disruption with particular emphasis on impaired function of astrocytes in ALS. We will discuss implication of membrane proteins expressed on astrocytic endfeet, aquaporin-4, and inwardly rectifying potassium channel in the pathology of ALS. In addition to ALS-specific IgGs, these membrane proteins are proposed as novel biomarkers of the disease.

## 1. Overview

Amyotrophic lateral sclerosis (ALS) is an adult-onset, progressive neurodegenerative disease that affects upper and lower motor neurons leading to fatal paralysis mostly within a few years. This devastating disease was described back in the 1870s, but despite the progress in biomedicine, no suitable therapeutic treatment has been introduced. Moreover, there is no distinguishable diagnostic test for the disease. Riluzole, a substance with antiglutamatergic properties, which modestly extends survival by 11% [1], remains the only approved drug for the treatment of ALS. The majority of research on ALS patients involves investigation of* postmortem* tissue samples. Development of* in vivo* approaches broadens the possibility to improve clinical research. In addition, identification of several different mutations that lead to ALS symptoms contributed to the generation of transgenic animal models widely used to examine mechanisms of ALS pathology [2–5].

ALS is in fact a complex disease with several proposed mechanisms, all of which lead to specific degeneration of motor neurons and the dysfunction of the neuromuscular junction. As one of the key mechanisms underlying the etiopathogenesis of ALS, glutamate excitotoxicity and its implications have been widely addressed and reviewed elsewhere. Here, we would like to point out some key features of brainstem and spinal motor neurons, important for their normal functioning that make them more susceptible in the elevated brain activity. Namely, ALS-vulnerable motor neurons have lower capacity for calcium buffering with larger calcium microdomains in the vicinity of open channels and high number of calcium permeable AMPA receptors. Importantly, these neurons depend considerably on surrounding astrocytes that are able to influence the expression level of the calcium impermeable GluR2 subunit of AMPA receptors and are responsible for glutamate clearance and potassium buffering (for reviews see [6–10]). However, there is a wealth of literature on aberrant populations of astrocytes in ALS. Astrocytes in ALS are known to change shape and swell [11, 12] that is indicative of their hampered extracellular ion filtering function [13]. On the other hand, there is evidence that in ALS astrocytes release toxic factors that are detrimental to motor neurons [14–18].

## 2. Neuroinflammation: A Focus on Humoral Immunity

Cellular markers of neuroinflammation in ALS (activated astrocytes and microglia, followed by infiltrated T-lymphocytes) are considered to be one of the main characteristics of ALS, as shown in numerous studies on human* postmortem* samples and on animal models of the disease (for reviews see [19–21]). All these findings suggest that neuroinflammation in ALS is not a temporary, but rather permanent, feature. The activation of the immune response, although beneficial at start, enters the vicious cycle that contributes to further neuronal damage, due to continuous presence of inflammatory cause, thus creating the inability to end the inflammatory reaction [19].

Over the last four decades, many attempts have been made to recognize useful biomarkers for ALS in biofluids. A concept of combining markers to improve accuracy has been widely supported, where some candidate biomarkers may be useful to aid in the diagnostics but may also have better applications in monitoring drug effects in clinical trials and as prognostic indicators of disease (for review see [22]). Here, we will focus on possible humoral immune markers, mainly circulating immune complexes (CICs) and immunoglobulins (Ig).

Several studies have demonstrated elevated CICs in ALS patients [23–26], but Saleh and coworkers (2009) were able to evaluate humoral immune response over disease progression by following the group of ALS patients in a 6-month period. They found elevated CICs as well as IgG in ALS sera in the initial disease stage, but, 6 months after the first tests, the same group of ALS patients, now with lower ALS functional rating score, had CICs lowered to the control levels, yet IgG remained higher than in controls [27]. Regarding the levels of IgG in ALS sera, literature data differ. Some report elevated IgG and normal IgM [26–29] with a change in subclasses [30] or without it [31], while others detect no change [25] or even decrease [32]. Inconsistency of reports is probably due to different selection criteria for ALS patients that were in different phases of the disease.

## 3. Specificity and (Patho)physiological Effects of ALS IgG

More than 20 years ago, Appel and coworkers showed that intraperitoneal injection of IgG purified from sera of sporadic ALS patients (ALS IgG) in mice increased the frequency of miniature end-plate potentials, without changing their amplitude, leading to increased acetylcholine release from motor nerve endings [33]. In addition, IgGs were detected inside motor neurons, as well as in end-plates. Taken together, ALS IgGs were able to elicit physiological abnormalities of nerve-muscle synapse by passive transfer, which indicated possible immune-mediated mechanisms in the pathogenesis of ALS. Passive transfer of ALS IgG in mice caused degeneration of motor neurons and increased proportion of calcium containing organelles [34]. In cell culture preparations, ALS IgG induced apoptotic cell death of the hybrid motoneuronal cell line VSC4.1 [35] and of the human neuroblastoma cell line [36]. In the rat mixed primary spinal cord cultures, ALS IgG activated caspase-3 and downstream signaling pathways, leading to selective apoptosis of neurons, while astrocytes were less susceptible; hence, the authors left an open possibility that neuronal death could be a secondary effect of the influence of ALS IgG on astrocytes [37].

Treatment of rodents (animals or isolated neuronal preparations) with ALS IgG caused increase in intracellular calcium concentration and degenerative structural changes as well as plasticity induction in the neuromuscular junction [34, 38–42]. Electrophysiological recordings of voltage-gated calcium currents showed an ALS IgG-induced increase in Purkinje neurons [43], but a decrease in cultured granular cells [44]. We have shown that ALS IgG induced the frequency increase, without amplitude changes of spontaneous miniature excitatory postsynaptic currents in primary rat hippocampal pyramidal neurons, and this effect was partially dependent on extracellular calcium [45]. Moreover, ALS IgG could modulate calcium transients in the same model system—an effect that was shown to be dependent on P/Q-type calcium channels [46].

In the recent study of Gonzalez and coworkers (2011) [47], a substantial evidence was presented in favor of the specificity of ALS IgG, as they addressed the distribution of autoantibodies via immunostaining in cerebellar, cortical, and spinal cord and neuromuscular junction sections of mice in addition to ALS IgG-induced physiological response via potentiating effect on end-plate basal discharge activity of neuromuscular junctions. Moreover, the use of Ca_V_2.1 knockout mice allowed them to distinguish between possible direct effect of ALS IgG on P/Q-type calcium channel (as congruent to our work; see above and [46]) and definite indirect (probably via phospholipase C) effect on N-type calcium channels [47].

The role of glial cells and their possible responses to ALS IgG was questioned in the study of the effect of these autoantibodies on motoneuronal morphology and survival in organotypic rat spinal cord cultures, where a range of concentrations of ALS IgG failed to induce significant changes [48]. Moreover, a brilliant cell-type sensitive genetic manipulation of the mouse model of ALS, that underlines the importance of neuron-glia interactions, where astrocytes and microglia are found to be implicated in the rate of progression of familial ALS, reported that the damage in astrocytes determines the timing of microglial activation and infiltration [49, 50]. Finally, our recent pioneer studies on ALS IgG-induced (patho)physiological changes in cultured rat cortical astrocytes detected specific acute astrocytic responses. Namely, the majority of ALS IgGs increased in a matter of minutes the mobility of acidic vesicles, mostly endosomes and lysosomes, which could suggest long-term modulation of endocytosis and/or autophagy [51]. In addition, half of ALS IgGs tested evoked transient increases in intracellular calcium, mediated via inositol-3-phosphate receptors in the endoplasmic reticulum, where the influx of calcium ions through store-operated calcium entry did not affect the amplitude but prolonged calcium transients, thus augmenting the overall magnitude of the response [52]. Elevated calcium could trigger the release of glutamate from astrocytes into the extracellular space via vesicular exocytosis [53], that could contribute to the excitotoxic loss of motor neurons. Although pharmacological analysis revealed activation of phosphatidylinositol 3-kinase upstream of phospholipase C, a direct ALS IgG target on astrocytes remains still elusive. The interaction of ALS-specific antibodies with an unknown astrocytic membrane structure, mediated via phosphatidylinositol 3-kinase that integrates pathways of cell growth, proliferation, differentiation, motility, survival, and intracellular trafficking and can interact with around 50 Src homology domains containing cellular enzymes [54], could have a great impact on etiopathogenesis of ALS, thus stressing out the urge for further investigations. On the other hand, these immune humoral factors also offer a possible new biomarker for ALS diagnostics. Nevertheless, although deposits of IgG were detected in* postmortem* spinal cords of ALS patients [55], until recently studies involving the interaction of disease specific antibodies and cells of immunologically privileged tissues were hampered by a lack of knowledge of the pathway of entry of immune factors in the CNS, particularly of the blood-brain (BBB) and the blood-spinal cord (BSCB) barrier that will be discussed in the following sections.

## 4. Blood-Spinal Cord and Blood-Brain Barrier Damage in ALS

First indications of disturbed permeability of BSCB and BBB in ALS patients came with the studies that examined blood and cerebrospinal fluid (CSF) composition in ALS patients. Thus, several immunological molecular markers of the BBB compromise were detected in blood and CSF [26]. More recent studies introduced several approaches for investigating BSCB and BBB condition in animal models of ALS, particularly in the regions of motor neuron degeneration. Intravenous application of Evans blue showed vascular leakage in spinal cord of end stage ALS animals [11, 12]. In ALS mice, permeability of vessels for Evans blue was observed even in early stage of disease [11]. Further examination was directed towards endothelial cells and their crucial role in control of transport across BSCB and BBB. Expression of tight junction proteins of endothelial cells, zonula occludens and occludin, was decreased in the spinal cord of ALS mice and rats [12, 56]. Investigation of BSCB and BBB ultrastructure using electron microscope showed swollen and degenerating endothelial cells and capillary rupture in early and end stage ALS mice [57]. Study of BSCB and BBB condition in the* postmortem* tissue of sporadic ALS patients confirmed findings on animal models [58]. These findings verified damage of endothelial cells and reduced expression of tight junction proteins and vascular leakage of IgG [58].

In order to explore the condition of BBB* in vivo*, the experiments on ALS rat brain were conducted using 1.5T magnetic resonance imager with surface coil. Application of gadolinium-DTPA contrast marker revealed increased BBB permeability in the brain of symptomatic ALS rat [21]. Simultaneously, antibodies against CD4+ and CD8+ receptors specifically labeled with ultrasmall superparamagnetic iron oxide particles (USPIO) were injected in order to follow infiltration of T-lymphocytes into the rat brain tissue using MRI. Accumulation of USPIO markers was detected in the tissue area surrounding lateral ventricles of the ALS rat. It was suggested that the infiltration of inflammatory cells in the ALS rat brain could be carried through the impaired BBB. A model proposed by Zlokovic (2008) [59] also indicated the BBB disruption as a crucial event in ALS pathogenesis. Based on the above studies, impairment of BBB opens the way for the entrance of inflammatory cells and factors into the CNS and leads to the activation of astrocytes and microglia and production of reactive oxygen species. These findings were also in favour of the studies of our group and others that showed the effect of ALS IgG on neurons and glia. Thus a hampered BBB or BSCB could offer the necessary pathway of entry for these immune factors (see above). Finally, this sequence of changes in the brain would result in a loss of function and death of motor neurons.

## 5. Involvement of Astrocytic Proteins in Blood-Brain Barrier Impairment

Although the most important components of BBB are endothelial cells, astrocytes contribute to BBB stability through perivascular endfeet ensheathing blood vessel wall. Moreover, astrocytic endfeet closely surround neuronal synapses contributing to the stability of neuronal microenvironment. Astrocytes express several proteins anchored in the membrane of their endfeet such as water channel aquaporin-4 (AQP4) and inwardly rectifying potassium channel Kir4.1 [60, 61]. These two transmembrane proteins colocalize and play a key role in the maintenance of water and ion homeostasis at the level of BBB and neuronal synapses [62, 63].

**Figure 1:**
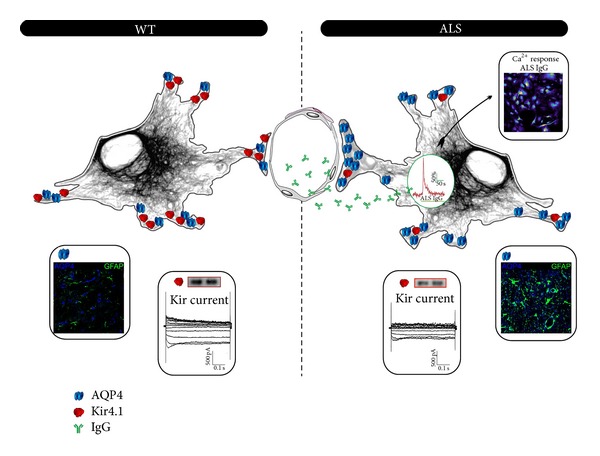
The molecular mechanism of ALS pathology in astrocytes. WT astrocyte (left) contains a balanced ratio of Kir4.1 and AQP4 channels in its cell membrane that also forms the endfeet around the endothelial layer of the blood vessel (center). ALS astrocyte (right) shows a misbalance of channel molecules with an abundance of AQP4 over Kir4.1. This causes swelling of endfeet and affects the BBB that becomes leaky for immune factors such as immunoglobulins (IgGs) that are known to cause intracellular calcium spikes (red trace and the pseudocolor image of a Ca^2+^-sensitive dye at the peak of the response in cultured astrocytes) and may start excitotoxic processes* in situ*. Colored panels in the bottom illustrate immunocytochemistry of AQP4 and GFAP in WT versus ALS astrocytes in the rat brain while the panels with traces illustrate whole-cell Kir currents from WT versus ALS astrocytes in culture and two examples of respective Western blot bands for Kir protein.

As astrocytes have an important role in the BBB maintenance, their involvement in molecular mechanisms underlying BBB disruption in ALS has been more thoroughly explored (Figure 1). Previous studies on animal models of ALS showed that astrocytic endfeet ensheathing the vessel wall appeared swollen indicating disturbance of water homeostasis and possible involvement of AQP4 [11, 12]. Our MRI investigations of the rat brain at end stage of ALS showed enlargement of lateral ventricles that further suggested impaired function of AQP4 [64]. For that reason, AQP4 expression was examined in several CNS regions affected in ALS. Recent studies have shown that AQP4 was overexpressed in the spinal cord, brainstem, and cortex of ALS rat [65, 66]. We have also documented that an increased expression of AQP4 was apparent in the astrocytic endfeet, particularly in those surrounding blood vessels [65]. Additionally, astrocytic endfeet that form glia limitans at the brain surface showed increased level of AQP4. Conversely, examination of Kir4.1 expression revealed a decrease in the spinal cord, brainstem, and cortex in ALS animal models [65, 67]. Moreover, in our studies increased AQP4 and reduced Kir4.1 expression in ALS were confirmed in cultured cortical astrocytes prepared from newborn SOD1 (G93A) rats [65]. These findings thus suggested novel molecular biomarkers for ALS and were in agreement with the changes observed in the brain tissue of symptomatic ALS rats. Our more detailed investigation revealed that functional properties of Kir4.1 channels were impaired in cultured ALS astrocytes. Thus, Kir currents isolated by addition of Ba^2+^ or Cs^+^ to the extracellular solution were reduced in ALS astrocytes. As also demonstrated by electrophysiology, exposure of cultured astrocytes to elevated concentrations of extracellular potassium showed reduced capacity in buffering excessive potassium ions in ALS [65]. Furthermore, it is known that increased extracellular potassium concentration causes death of motor neurons* in vitro *[67]. Decreased ability of ALS astrocytes to take excessive potassium ions released after neuronal activity could lead to a disturbance of ion balance in the vicinity of motor neurons and subsequently to their degeneration.

## 6. Conclusion

Neuroinflammation is particularly prominent in ALS. It was shown that ALS IgG along with immune T cells could enter the brain parenchyma via a hampered BBB thus affecting neurons as well as astrocytes. Thus ALS IgG could augment excitotoxicity by stimulating transmitter release in neurons, intracellular calcium transients in neurons, and glia and vesicle trafficking/exocytosis in astrocytes. This may also confirm ALS IgG as a potential humoral marker of the disease. On the other hand it has been shown that the BBB compromise may lie in the misbalance of expression of the two membrane channels of the astrocytic endfeet, also possible ALS biomarkers, Kir4.1 and AQP4.
